# Unveiling the nanotoxicology of snake venoms through functional and biochemical characterization of extracellular vesicles from *Naja naja* and *Daboia russelii*

**DOI:** 10.1038/s41598-025-27041-6

**Published:** 2025-11-29

**Authors:** Nagendra K, Shankar M. Bakkannavar, Vinutha R. Bhat, Freston Marc Sirur

**Affiliations:** 1 Department of Forensic Medicine and Toxicology, Kasturba Medical College, Manipal, Manipal Academy of Higher Education, Manipal, 576104 India; 2 Department of Biochemistry, Kasturba Medical College, Manipal, Manipal Academy of Higher Education, Manipal, 576104 India; 3 Department of Emergency Medicine, Kasturba Medical College, Manipal, Manipal Academy of Higher Education, Manipal, 576104 India

**Keywords:** Snake venom, Snake venom extracellular vesicles, Coagulation assays, Hemolysis, ATPase/ADPase activity, Venom toxicology, Biochemistry, Chemical biology, Drug discovery, Physiology

## Abstract

Snake venom is a complex biochemical arsenal that exerts multifaceted effects on host physiology, primarily targeting the hemostatic, cellular, and metabolic systems. Recent attention has turned to venom-derived extracellular vesicles (EVs) as active carriers of toxin components. The current study comparatively evaluates the hematological and enzymatic effects of crude venoms and EVs from *Naja naja* and *Daboia russelii* across key physiological parameters: prothrombin time (PT), activated partial thromboplastin time (aPTT), red blood cell hemolysis, and purinergic enzyme activity (ATPase and ADPase). Both venoms and their EVs caused dose-dependent alterations in coagulation, with EVs significantly prolonging aPTT and PT, indicating intrinsic and extrinsic pathway inhibition. Hemolysis assays revealed sex-specific erythrocyte susceptibility, with higher rates observed in female samples. Additionally, venom-derived EVs modulated ATPase and ADPase activity, interfering with cellular energy metabolism and thromboregulation. Our findings establish venom EVs as potent functional units that replicate and amplify native venom toxicity. These results underscore the importance of targeting EV-associated toxins in antivenom development and highlight the evolving complexity of venom systems biology. This study broadens our understanding of venom pathophysiology and identifies novel diagnostic and therapeutic avenues against snakebite envenomation.

**Supplementary Information:**

The online version contains supplementary material available at 10.1038/s41598-025-27041-6.

## Introduction

Snakebite envenoming is a significant yet often overlooked global public health issue, predominantly affecting rural populations in tropical regions. Approximately 5.4 million snakebites occur annually worldwide, leading to 1.8–2.7 million cases of envenomation and between 81,410 and 137,880 deaths^1^.

The burden is especially heavy in Sub-Saharan Africa, South America, and Asia, where limited access to healthcare exacerbates the impact. India bears a substantial portion of this burden, with an estimated 58,000 deaths due to snakebites each year^2,3^. Significant snake fauna found in India includes several venomous species, notably the “Big Four”, namely the Indian cobra (*Naja naja*), common krait (*Bungarus caeruleus*), Russell’s viper (*Daboia russelii*), and the saw-scaled viper (*Echis carinatus*). Among the Big Four, the Russell’s viper and Indian cobra are responsible for the highest number of snakebite cases in India among the *Viperidae* and *Elapidae* families, respectively^2^. These species are responsible for most of the envenomation and fatalities. The interaction between humans and snake species, especially in rural areas, heightens the risk of snakebites. Efforts are underway to address this challenge; the World Health Organisation aims to reduce snakebite-induced deaths and disabilities by 50% by 2030^4^.

Despite these initiatives, underreporting remains a significant obstacle, often due to socio-economic and cultural factors that lead victims to seek traditional treatments over medical care. Accurate data collection, public education, and improved access to healthcare are crucial steps toward mitigating the impact of snakebite envenoming.

Snake venom composition varies between the families, In case of Viperidae snake venom, enzymatic proteins makes up 80–90% of venom dry weight; major enzyme includes, Phospholipase A2 (PLA2), Metalloproteases, Serine proteases, L-amino acid oxidase (LAAO), Hyaluronidase, Nucleases, Arginine ester hydrolases and Non-enzymatic proteins constitutes 10–20% of venom, which Includes Disintegrins, C-type lectins, CRISPs, natriuretic peptides, nerve growth factors, proteinase inhibitors, bradykinin-potentiating peptides. Whereas Elapidae snake venom’s enzymatic proteins constitute 25–70% of venom dry weight, lower than Viperidae venom, major enzyme includes PLA2, Hyaluronidase, nucleases, metalloproteases, serine proteases and non-enzymatic protein ranges from 30 to 75% of venom, including Three-finger toxins (3FTx), Kunitz-type serine protease inhibitors, natriuretic peptides, bradykinin-potentiating peptides, nerve growth factors, proteinase inhibitors but 3FTx are dominant^5^. The composition of the snake venom varies based on various factors, such as the snake’s gender, geographical location, diet, age, environmental factors, and evolutionary adaptations^6^. Snake venom components are formed inside the snake’s venom glands as pre-pro proteins, which have signal peptides. During maturation, the proteins lose the signal peptides and remain soluble within the aqueous venom matrix.

A study on the inferred amino acid sequences from the cloned cDNAs of aminopeptidase A (APA) and dipeptidyl peptidase IV (DPP IV) from the venom gland of *Gloydius blomhoffi brevicaudus* showed that both proteins are type II membrane proteins with a single transmembrane domain close to the N-terminus. Analysis of the N-terminal amino acid sequence of purified DPP IV revealed an unprocessed N-terminal^7^. This unique molecular feature sparked curiosity about the mechanism of protein secretion into venom. One possible pathway involves extracellular vesicles (EVs), which are secreted by various cell types and can transfer proteins, lipids, and other biomolecules to recipient cells.

### Exosomes and microvesicles are classes of EVs

Exosomes are formed by the invagination of the Endosomal membrane, leading to multiple vesicles inside the endosomes, known as multivesicular bodies (MVBs). These MVBs fuse with the plasma membrane and release their internal vesicles into the extracellular environment. Meanwhile, microvesicles are formed by the outward growth and fission of the plasma membrane.

EVs are produced by various sources, from microbes to humans, using a variety of body fluids like blood, saliva, urine, sweat, milk, and semen. In general, EVs are involved in various biological processes, from cell signalling to immunological response, in some pathological conditions such as cancer, CVDs, infectious diseases, and neurological diseases.

Presence of EVs in the snake venom was initially observed in the venom glands of American rattlesnakes^8^. Comparative characterisation of the vesicles based on their biochemical activity has not been done extensively.

The current study compares the biochemical activities between the *Naja naja* and *Daboia russelii* snakes’ venom and their EVs.

## Materials and methods

### Snake venom sampling permits

**Fig. 1:**
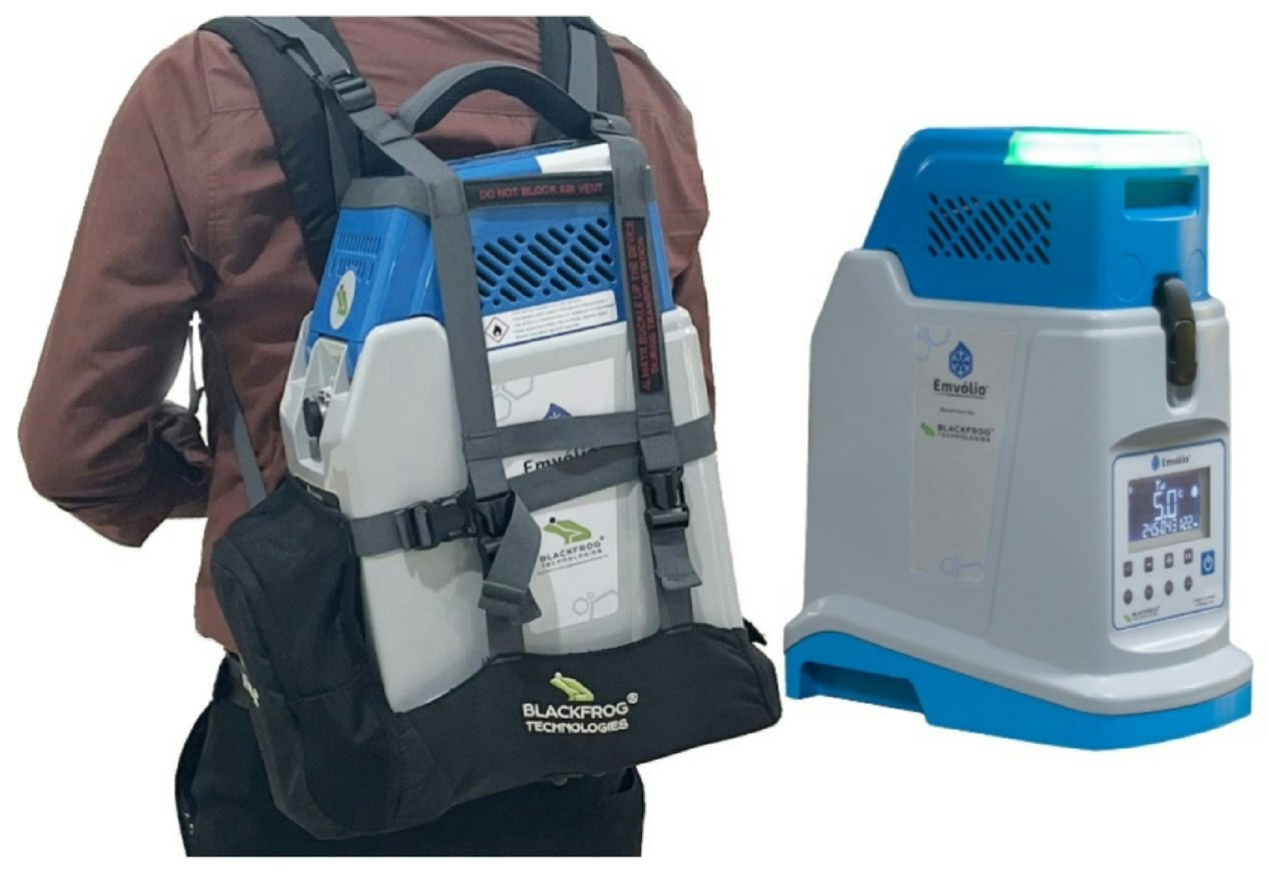
Portable active cooled carrier for biological samples.

After obtaining the permission from the Karnataka State Forest Department, (Letter no. E-769017 KFD/WL/E2/16/2022-WL,16.12.2022), expert herpetologist assigned by the Forest department identified the snake and collected the venom samples from 9 *Naja naja* snakes and 6 *Daboia russelii* in accordance with WHO protocol; samples were pooled and transported to the study center using – 20 °C (Fig. 1) a portable active cooled carrier for transport of biologicals; designed by Blackfrog technologies, Manipal^9^. At the study centre, samples were stored in the − 80 °C freezer.

### Ethical permissions

Ethical clearance was obtained from the Institute’s Human Ethics Committee, Kasturba Medical College, Manipal (IEC1: 36/2022, 09/02/2022) and from the Institutional Biosafety Committee, Manipal Academy of Higher Education, Manipal, prior to conducting the experiments. Blood samples were collected from healthy individuals, aged 18–40 years, via the median cubital vein using a 21-gauge needle after obtaining written consent.

### Isolation and characterisation of extracellular vesicles

#### Isolation of the extracellular vesicles

EVs were separated by ultracentrifugation as proposed by Souza-Imberg et al., with minor changes, especially in centrifugation time^10^. Crude venom samples of *Naja naja* and *Daboia russelii* were diluted in a 1:4 ratio in phosphate buffer. The diluted venom samples were centrifuged for 30 min at 15,000 × g at 4 °C to discard cell debris and bigger particles. The resulting supernatants were then ultracentrifuged at 120,000 × g for 2 h at 4 °C using a swinging bucket rotor (SureSpin 630/17) in a Sorvall™ WX 90 + ultracentrifuge (Thermo Scientific, USA).

The ultracentrifugation pellets containing EVs were split into two. One was resuspended in phosphate buffer for NTA, while the other pellet was resuspended in RIPA buffer with a protease inhibitor cocktail for other biochemical tests. The identity and integrity of the isolated EVs were confirmed as per the MISEV (Minimal Information for Studies of Extracellular Vesicles) guidelines^11^.

#### Nanoparticle tracking analysis

To determine the size distribution and concentration of the vesicles, Nanoparticle tracking analysis was done. Pellets containing *Naja Naja* and *Daboia russelii* EVs were resuspended in PBS (pH 7.4) and used for the Nanoparticle tracking analysis using NanoSight LM10 (Malvern Instruments, UK). Samples were diluted accordingly with sterile PBS to obtain the result.

#### Transmission electron microscopy (TEM)

The ultrastructure of the isolated EVs was determined by transmission electron microscopy. In brief, the EVs sample was diluted in a 1:5 ratio, and a few drops of the suspension of EVs were placed onto 300 mesh carbon-coated grids at room temperature. After five minutes, the excess fluid was removed by blotting with filter paper and washed twice with distilled water. The grids coated with carbon were stained by continuous dripping of 0.5% uranyl acetate, and the excess solution was blotted and air-dried for 10 min at room temperature. Images were collected using a Jeol 120 kV transmission microscope.

#### Protein estimation

The Bicinchoninic acid Method was used to estimate the protein concentration for the *Naja naja* and *Daboia russellii* snakes’ venom and lysed snake venom extracellular vesicles (SVEVs). Briefly, 25 µl of varying concentrations of BSA (25–2000 µg/ml) was used as standards along with the diluted Snake venom, SVEVs and mixed with 200 µl of BCA reagents in a 96-well plate and incubated at 37 °C for 30 min. Intensity of the colour was measured at 562 nm^12^.

#### Evaluation of extracellular vesicle-specific marker expression

Western blotting confirmed the expression of EV-specific markers such as TSG101, CD63 and negative marker HSP 90β. A total of 15 µg of protein from snake venom and snake venom extracellular vesicles (SVEVs) was loaded into each well of a 12% SDS-PAGE gel. Electrophoretic separation was performed at a constant voltage of 50 V for 150 min. After the successful separation, the protein bands were blotted to the PVDF membrane, and the blotting was confirmed through the Ponceau staining. Blots were incubated with primary antibodies (CD63, TSG101, HSP 90β) for 12–16 h at 4 °C. After that, membranes were incubated with secondary antibody for around 1 h and 15 min at room temperature. The protein was detected with chemiluminescent reagent and was imaged using the Gel Documentation system.

### Biochemical analysis

#### Acetylcholine esterase activity assay

Esterase activity was measured according to Ellmann’s method^13^. SVEV’s lysate was prepared manually on ice and centrifuged to remove the debris. The supernatant was used for the AChE assay after estimating the protein concentrations. Substrate acetylthiocholine was incubated with 5,5′-dithiobis-(2-nitrobenzoic acid) at 25 °C for 10 min, after which various concentrations(1, 5, 10, 20, 40 µg/ml) of *Naja naja* and *Daboia russelii* snake venom and SVEVs were mixed in different tubes containing the above reaction mixture and readings were measured immediately in a kinetic mode for 5 min at 412 nm using Genesys 50 (thermos scientific) spectrophotometer.

#### Prothrombin time and activated partial thromboplastin time

To quantify the ability of *Naja naja* and *Daboia russelii* venom and their EVs to alter the extrinsic and intrinsic coagulation pathways, the PT and aPTT, respectively, were performed. Healthy volunteers’ blood samples were collected in citrated vacutainer (*n* = 10, 4 female and 6 male), centrifuged at 3000 × g for 10 min at 4 °C to collect the platelet poor plasma (PPP). Prothrombin time was determined by incubating the PPP for 3 min with 5, 10, 20, 40 µg/ml concentrations of *Naja naja* and *Daboia russelii* snake venom and SVEVs in different tubes. The concentration range was selected based on previous reports^6^. After the incubation, pre-warmed PT reagent was added, and the time required to form the clot was noted (Agappe Diagnostic Ltd, India). For aPTT determination, PPP was incubated with activated cephaloplastin reagent, and at the end, CaCl2 was added, and the time required to form the first clot was noted (Agappe Diagnostic Ltd, India). In both PT and aPTT assays, a PBS was used as a buffer control to rule out any effect of citrate or reagents on clotting time.

#### Rate of hemolysis

Hemolytic activity of various concentrations of *Naja naja* and *Daboia russelii* snake venom and SVEVs was determined using human red blood cells using a previously described protocol with slight modification^14^. Whole blood samples from the healthy individuals (*n* = 10, 4 females and 6 males) were collected in a heparinised tube. Plasma and buffy coat were removed by centrifuging at 4500 rpm for 15 min at 4 °C, and the RBCs were washed with PBS and centrifuged, and the supernatant was discarded. The procedure was repeated until a clear supernatant was obtained. 1% RBC suspension was prepared and mixed with various concentrations (5, 10, 20, 40 µg/ml) of snake venom and SVEVs, incubated for 24 h at 37 °C. Post 24 h reaction mixtures were centrifuged at 4500 rpm for 10 min, and the absorbance of the supernatant was measured at 540 nm using a Multiscan SkyHigh (Thermo Scientific) microplate spectrophotometer. Triton X was used as a positive control.

#### ATPase and adpase assay

To determine the snake venom and SVEV’s ability to hydrolyse ATP and ADP, ATPase and ADPase assays were performed. 34 µg each of *Naja naja* and *Daboia russelii* snake venom and SVEVs were incubated at 37 °C along with incubation buffer (1 mg/ml ATP or ADP in 50 mM Tris-Cl and 3.8 mM MgCl_2,_ pH 7.4). The concentration of venom and SVEV was selected based on previous reports^6^. Tests were done in 2 sets for 1- and 2-h incubation. Following incubation, the colouring reagent (10% Ascorbic acid and 0.42% Ammonium molybdate in 1 N H_2_SO_4_) was added, and the mixtures were microwaved for 30 s at maximum potency. Trichloroacetic acid (0.3 M) was added to stop the reaction. Absorbance was measured at 820 nm using a Multiscan SkyHigh (Thermo Scientific) microplate spectrophotometer. All the tests were carried out in triplicate^15^.

### Statistical analysis

Descriptive statistics have been presented as mean and standard deviation. One-way ANOVA and Two-way ANOVA were employed for group comparison using GraphPad Prism Software 10.0^16^.

## Results

### Characterisation of extracellular vesicles

The isolation of EVs from the crude non-lyophilised venom from the *Naja naja* and *Daboia russelii* was done using the ultracentrifugation technique.

**Fig. 2:**
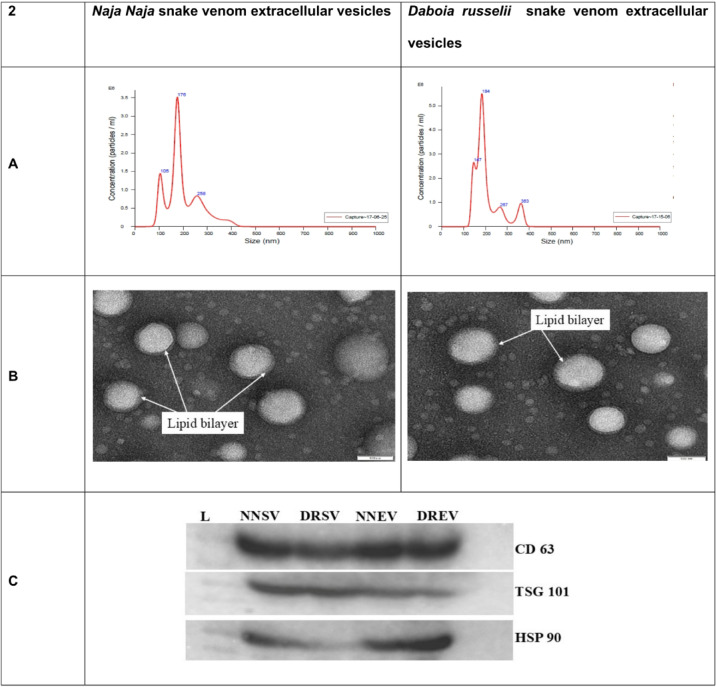
Characterisation of snake venom extracellular vesicles: (**A**) represents the size distribution of isolated EVs demonstrated by Nano sight LM10 for *Naja naja* and *Daboia russelii* snakes’ venom extracellular vesicles. (**B**) Represents TEM images of isolated *Naja naja* and *Daboia russelii* snakes’ venom extracellular vesicles. (**C**) Represents the western blotting of *Naja naja* Snake Venom (NNSV), *Daboia russelii* snake venom (DRSV), *Naja naja* snake venom extracellular vesicles (NNEV), and *Daboia russelii* snake venom extracellular vesicles (DREV), which are positive for EV cytoplasmic marker TSG 101 and EV transmembrane marker CD 63, partially positive for HSP90, and endoplasmic reticulum marker.

The pellet was resuspended in Phosphate buffer to validate the isolated EVs and subjected to Nanoparticle Tracking analysis (NTA) and Transmission Electron Microscopy (TEM). NTA analysis showed the mean EV size present in the *Naja naja* venom was 199.1 nm, whereas in the *Daboia russelii* venom, EVs were 207 nm (Fig. 2A). The ultrastructure of the EVs under TEM revealed the double-layered membranes and oval particles (Fig. 2B). NTA revealed a broader and polydisperse distribution of EV sizes compared to Transmission Electron Microscopy. Western blotting showed the presence of CD63, TSG101 and HSP 90β in the isolated EVs of both *Naja naja* and *Daboia russelii* venom (Fig. 2C). HSP 90β was found to be positive, which may be due to the stress condition of the captured snakes reflected in the venom or may be co-isolated with the EVs.

#### Protein estimation

Protein concentration in the *Naja naja* and *Daboia russelii* snake venom and their SVEVs was estimated by the BCA method and given in Table 1.

**Table 1:** Protein concentrations in snake venom and their extracellular vesicles (tests were carried out in triplicate).

Species name	Snake venom protein concentrations	Snake venom extracellular vesicles protein concentrations
*Naja naja*	135 mg/ml	15 mg/ml
*Daboia russelii*	139 mg/ml	08 mg/ml

### Biochemical analysis

#### Acetylcholine esterase activity assay

**Fig. 3:**
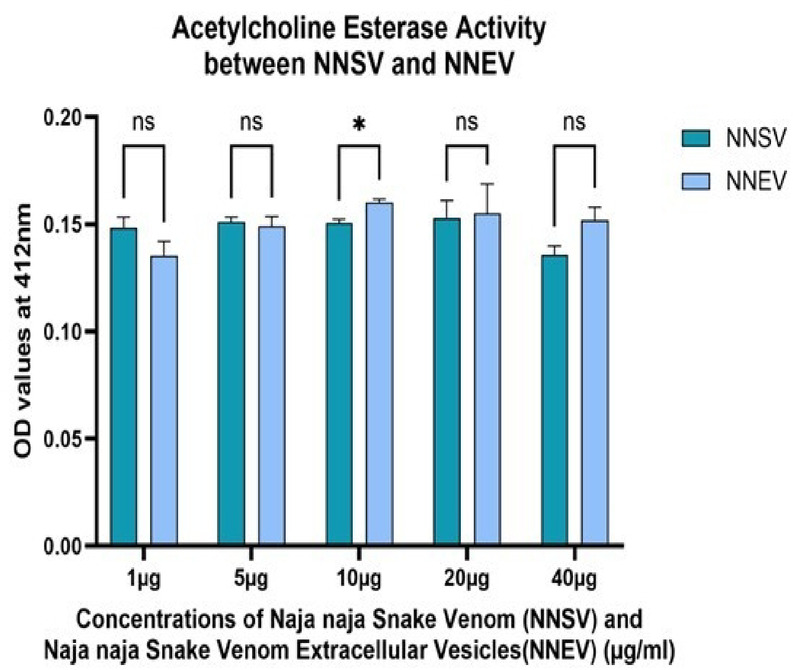
Acetylcholine esterase activity assay among *Naja naja* snake venom (NNSV) and *Naja naja* snake venom-derived extracellular vesicles (NNEV). All assays were performed in triplicate, and the standard deviation is indicated by error bars. Statistical significance is indicated using the conventional asterisk system: *p < 0.05, **p < 0.001, ***p < 0.0001 and ****p < 0.0001.

**Fig. 4:**
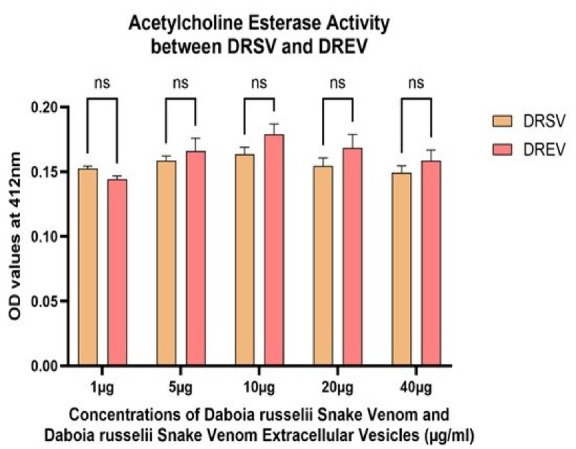
Acetylcholine esterase activity assay among *Daboia russelii* snake venom (DRSV) and *Daboia russelii* snake venom-derived extracellular vesicles (DREV). All assays were performed in triplicate, and the standard deviation is indicated by error bars. Statistical significance is indicated using the conventional asterisk system: *p < 0.05, **p < 0.001, ***p < 0.0001 and ****p < 0.0001.

**Fig. 5:**
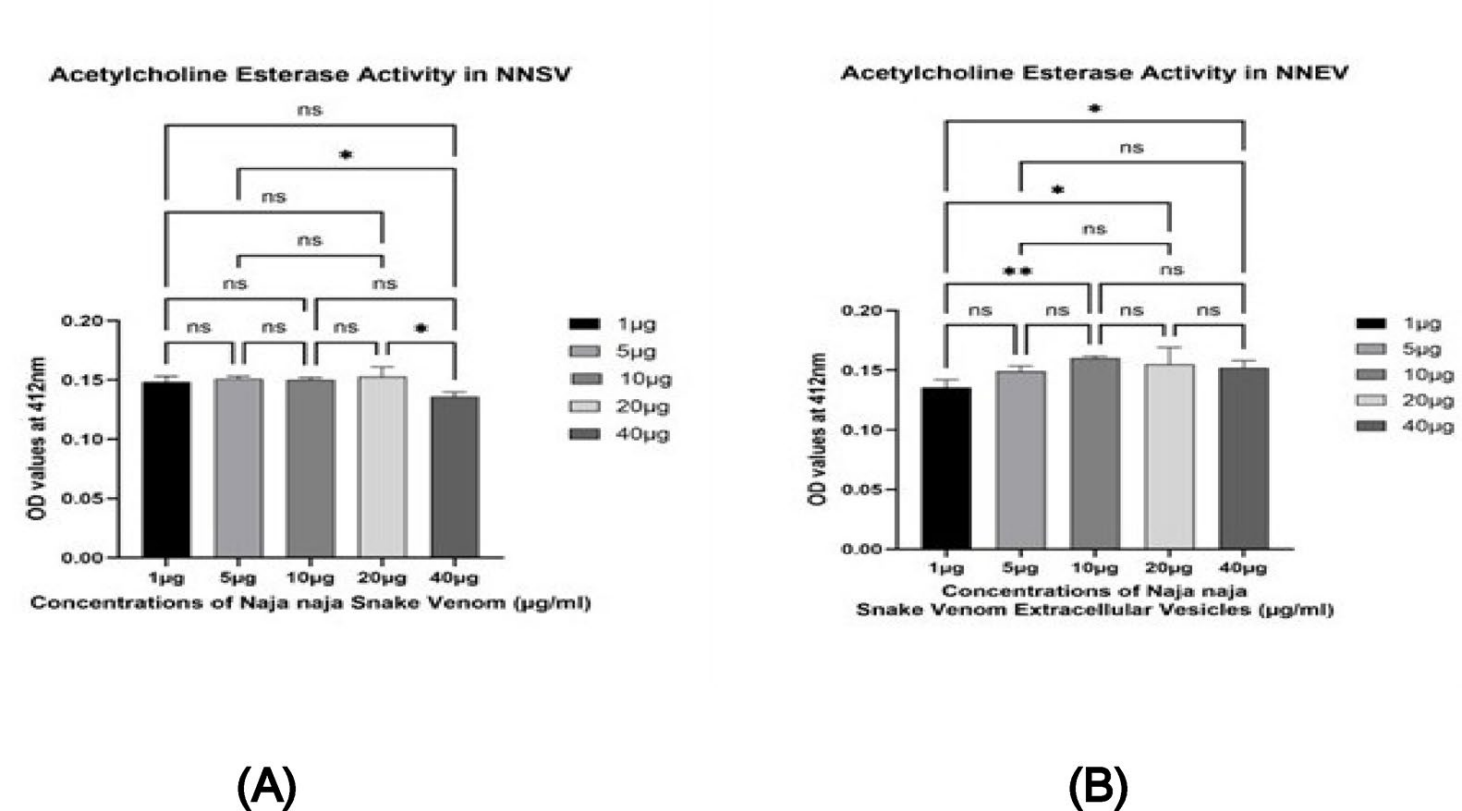
(**A**) Different doses of Acetylcholine esterase activity of *Naja naja* snake venom (NNSV). All assays were performed in triplicate, and the standard deviation is indicated by error bars. Statistical significance is indicated using the conventional asterisk system: *p < 0.05, **p < 0.001, ***p < 0.0001 and ****p < 0.0001. (**B**) With varying doses, acetylcholine esterase activity of *Naja naja* snake venom-derived extracellular vesicles (NNEV). All assays were performed in triplicate, and the standard deviation is indicated by error bars. Statistical significance is indicated using the conventional asterisk system: *p < 0.05, **p < 0.001, ***p < 0.0001 and ****p  < 0.0001.

**Fig. 6:**
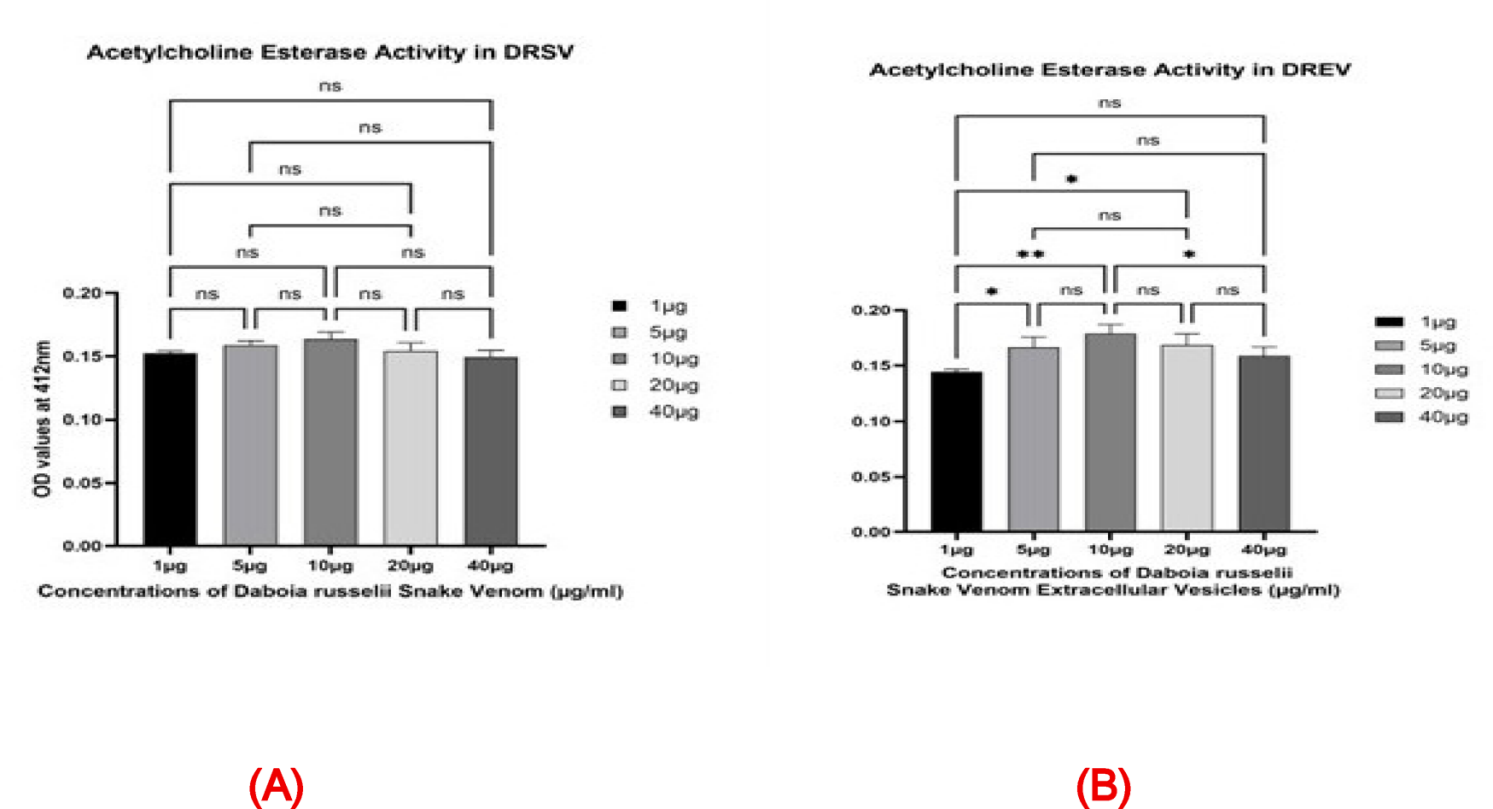
(**A**) Acetylcholine esterase activity among Daboia russelii snake venom (DRSV) with varying doses. All assays were performed in triplicate, and the standard deviation is indicated by error bars. Statistical significance is indicated using the conventional asterisk system: *p < 0.05, **p < 0.001, ***p < 0.0001 and ****p < 0.0001. (**B**) Acetylcholine esterase activity among Daboia russelii snake venom-derived extracellular vesicles (DREV) with varying doses. All assays were performed in triplicate, and the standard deviation is indicated by error bars. Statistical significance is indicated using the conventional asterisk system: *p < 0.05, **p < 0.001, ***p < 0.0001 and ****p < 0.0001.

Figure 3 the graph illustrates the acetylcholine esterase (AChE) activity in *Naja naja* snake venom (NNSV) and its venom-derived extracellular vesicles (NNEV) across varying concentrations (1 µg,5 µg, 10 µg, 20 µg, and 40 µg). Optical density (OD) values were recorded at 412 nm, indicating the enzymatic hydrolysis of acetylcholine by AChE.

From Fig. 3, at the lowest concentration (1 µg), NNSV exhibited slightly higher AChE activity than NNEV. From 5 to 40 µg, NNEV consistently showed equal or marginally higher OD values than NNSV, suggesting equal or comparable AChE activity in the extracellular vesicles, though statistically nonsignificant when compared between NNSV and NNEV across the concentration, except 10 µg (p value 0.01). Meanwhile, NNSV and NNEV showed comparable AChE activity, indicating that venom-derived extracellular vesicles retain or enhance the enzymatic function of the whole venom. NNSV and NNEV showed a positive correlation between concentration and AChE activity up to 10 µg, followed by a plateau, consistent with enzyme saturation kinetics (Fig. 4).

In case of NNSV, AChE activity was almost consistent up to 20 µg among its doses, and decreased at 40 µg [p value 0.02 between 20 and 40 µg (Fig. 5a)]. A dose-dependent increase in NNEV AChE activity from 10 µg when compared to the lowest concentration (1 µg vs. 10 µg, p value 0.004; 1 µg vs. 20 µg, p value 0.016; 1 µg vs. 40 µg, p value 0.039) (Fig. 5b), after which the activity starts decreasing.

From Fig. 4, at the lowest concentration (1 µg), DRSV exhibited slightly higher AChE activity than DREV. From 5 µg to 40 µg, DREV consistently showed equal or marginally higher OD values than DRSV. However, it is nonsignificant, suggesting equal or comparable AChE activity in the extracellular vesicles.

Though AChE activity in DRSV and the DREV counterparts positively correlated with concentration and comparable activities, DREV displayed slightly higher activity from 5 to 40 µg concentrations when compared to DRSV (Fig. 4). This indicates that venom-derived extracellular vesicles retain or enhance the enzymatic function of the whole venom.

From graph 6A, DRSV AChE activity was slightly higher at 10 µg when compared to the doses on either side visually, but not statistically significant (Fig. 6a). A dose-dependent increase in DREV AChE activity up to 10 µg when compared with the initial concentration 1 µg (1 µg vs. 5 µg, p value 0.02; 1 µg vs. 10 µg, p value 0.001 (Fig. 6b)) after which the activity starts decreasing.

### Prothrombin time and activated partial thromboplastin time

**Fig. 7:**
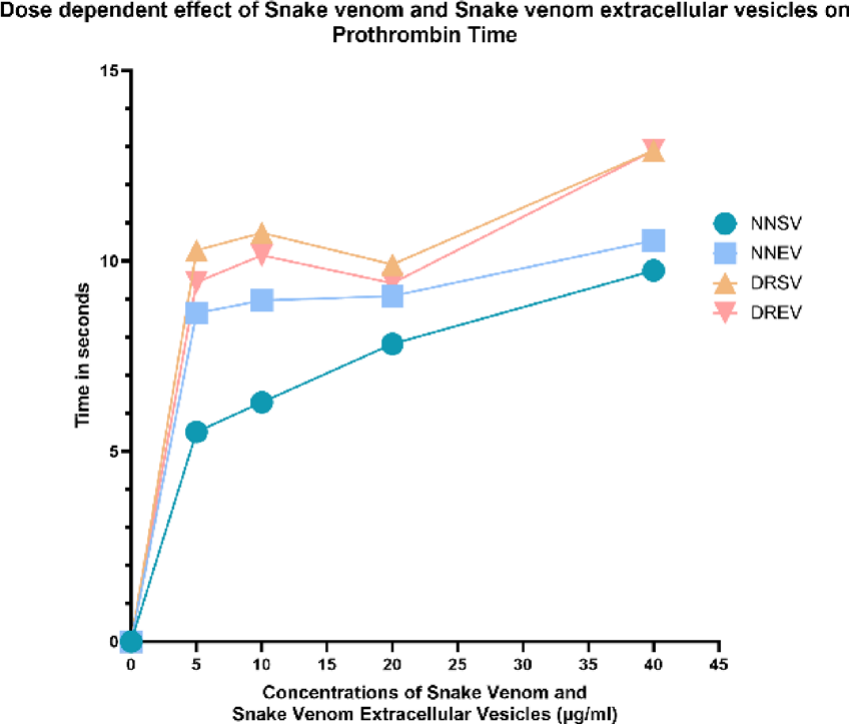
The graph illustrates the PT responses to varying concentrations of the four venom components: *Naja naja* venom (NNSV), *Naja naja* EVs (NNEV), *Daboia russelii* venom (DRSV), and *Daboia russelii* EVs (DREV). All assays were performed in triplicate.

**Fig. 8:**
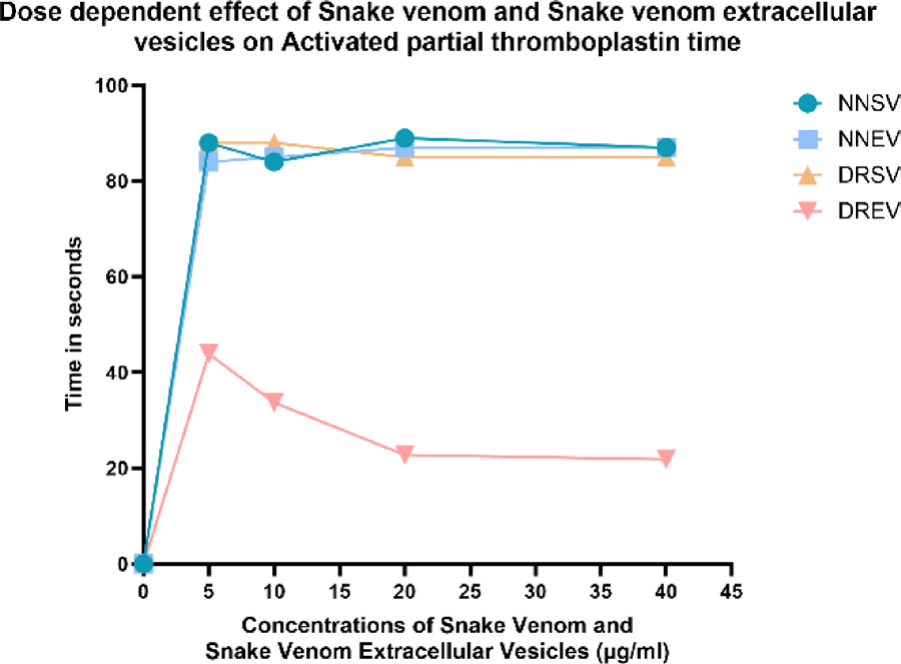
The graph illustrates the aPTT responses to varying concentrations of the four venom components: *Naja naja* venom (NNSV), *Naja naja* EVs (NNEV), *Daboia russelii* venom (DRSV), and *Daboia russelii* EVs (DREV). All assays were performed in triplicate.

NNSV demonstrated a gradual, dose-dependent increase in PT, indicating a consistent anticoagulant effect with rising concentrations. NNEV showed a sharp initial increase in PT at low concentrations, followed by a plateau, suggesting a potent anticoagulant effect that stabilises beyond 5 µg concentration. DRSV exhibited a similar pattern to NNEV, with a rapid increase in PT at lower concentrations, after which its activity exhibits a plateau till a 20 µg dose; thereafter, the anticoagulation effect continues to show dose-dependent intensification. DREV displayed the most significant prolongation of PT at the highest concentration tested, indicating a strong anticoagulant effect that intensifies with increasing doses (Fig. 7).

aPTT is at baseline at 0 s, as the concentration increases from 5 to 20 µg/mL, NNSV, NNEV, and DRSV show a steep increase in aPTT to approximately 85–90 s at 5 µg/mL. Further increase in venom concentrations did not show concomitant prolongation of aPTT, with concentrations beyond 5 µg/mL (10–20 µg/mL) showing a plateau (no change) in aPTT. This indicates that the maximum anticoagulant effect is achieved early and sustained. Unlike the others, DREV shows a substantially lower increase in aPTT at all concentrations. At 5 µg/mL, aPTT reaches only ~ 45 s. As the concentration increases to 20 µg/mL, aPTT decreases progressively to ~ 25 s, suggesting a dose-dependent procoagulant effect (Fig. 8).

### Rate of hemolysis

**Fig. 9:**
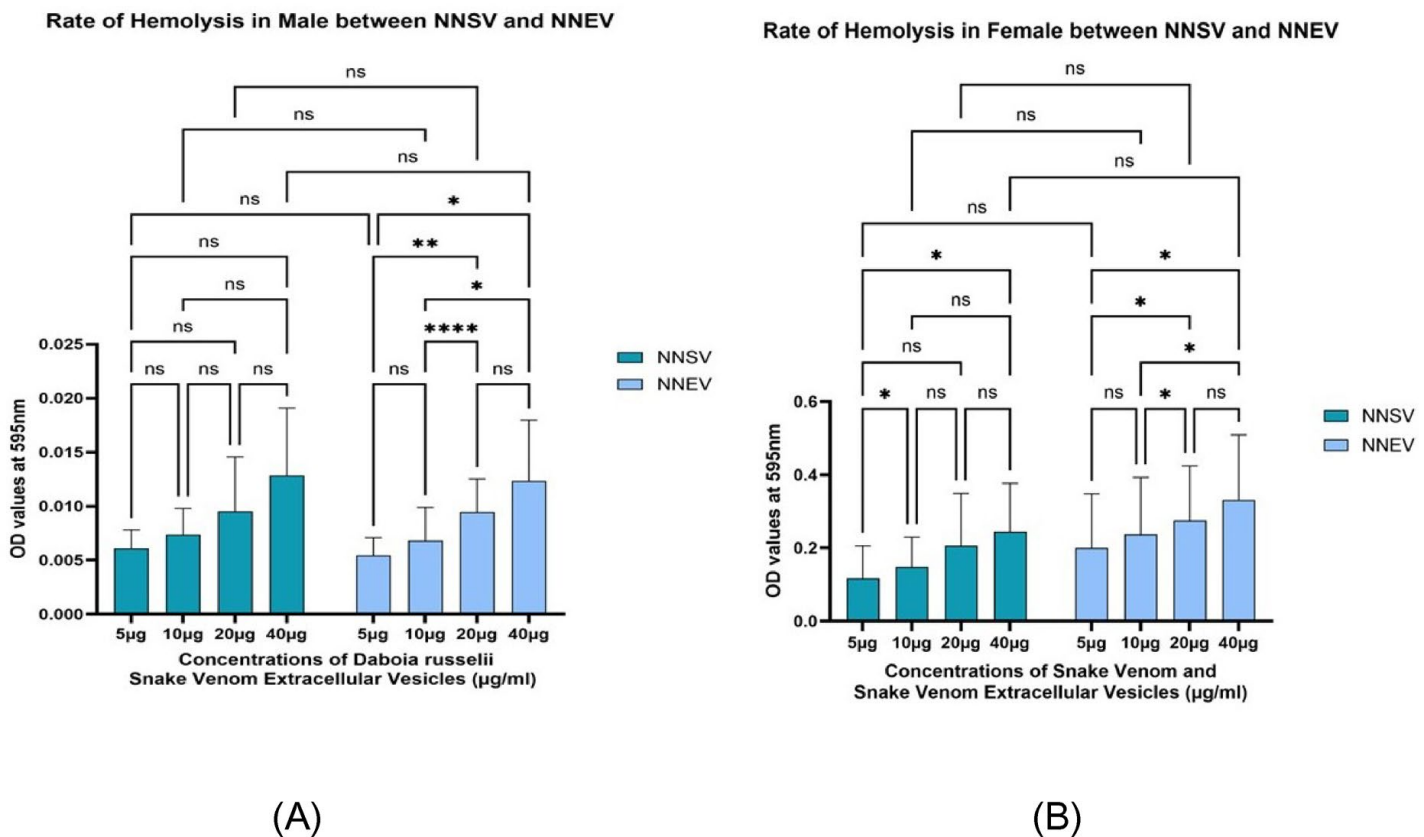
(**A**) The graph illustrates the hemolysis rate in males, based on the varying concentrations of *Naja naja* snake venom (NNSV) and *Naja naja* EVs (NNEV). All assays were performed in triplicate, and the standard deviation is indicated by error bars. Statistical significance is indicated using the conventional asterisk system: *p < 0.05, **p < 0.001, ***p < 0.0001 and ****p < 0.0001. (**B**) The graph illustrates the hemolysis rate in females, based on the varying concentrations of *Naja naja* snake venom (NNSV) and *Naja naja* EVs (NNEV). All assays were performed in triplicate, and the standard deviation is indicated by error bars. Statistical significance is indicated using the conventional asterisk system: *p < 0.05, **p < 0.001, ***p < 0.0001 and ****p < 0.0001.

**Fig. 10:**
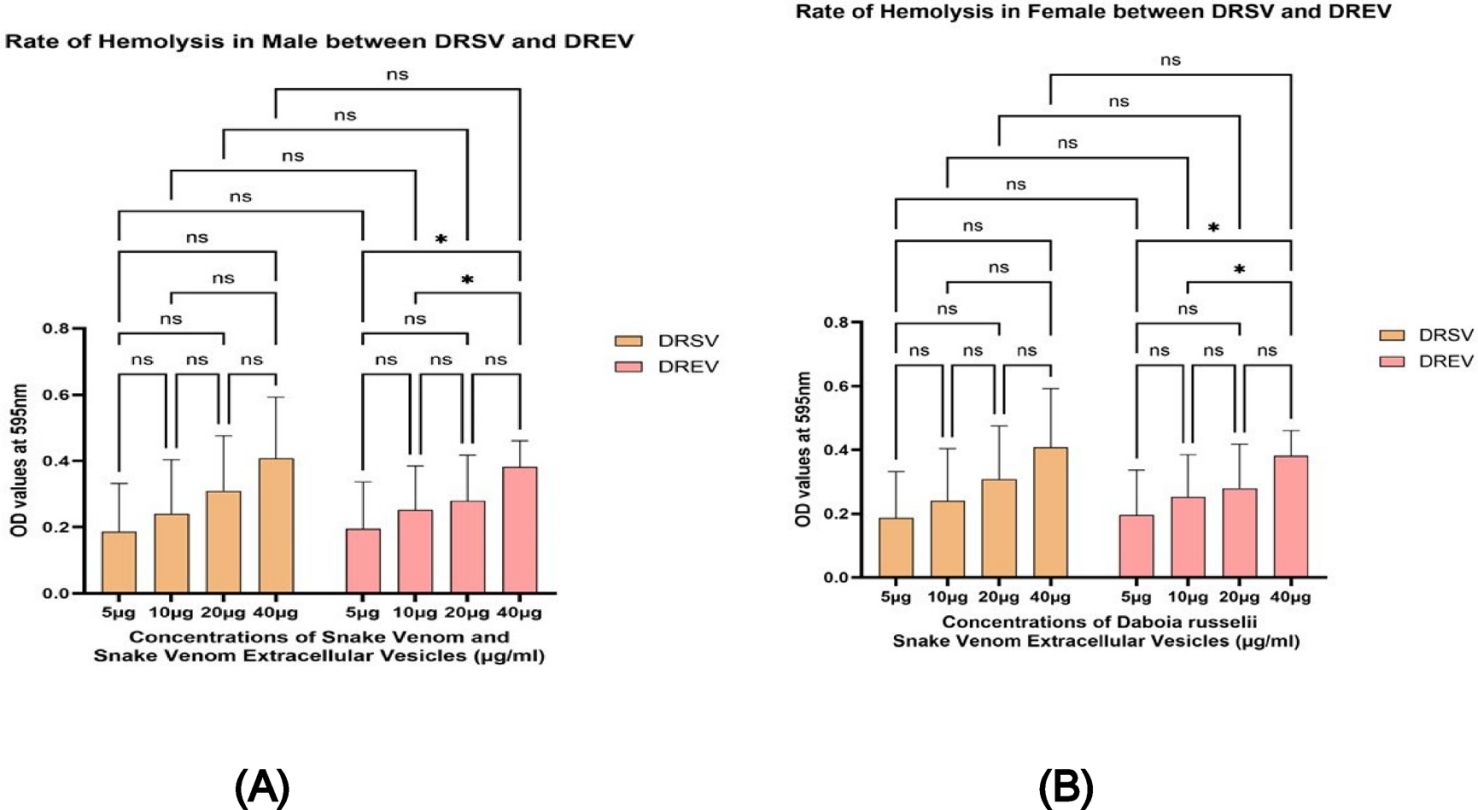
(**A**) The graph illustrates the hemolysis rate of the varying concentrations of the *Daboia russelii* snake venom (DRSV) and *Daboia russelii* EVs (DREV)in Males. All assays were performed in triplicate, and the standard deviation is indicated by error bars. Statistical significance is indicated using the conventional asterisk system: *p < 0.05, **p < 0.001, ***p < 0.0001 and ****p < 0.0001. (**B**) The graph illustrates the hemolysis rate of the varying concentrations of the *Daboia russelii* snake venom (DRSV), and *Daboia russelii* EVs (DREV) in males. All assays were performed in triplicate, and the standard deviation is indicated by error bars. Statistical significance is indicated using the conventional asterisk system: *p < 0.05, **p < 0.001, ***p < 0.0001 and ****p < 0.0001.

Rate of Hemolysis in case of females, NNSV displayed the lowest hemolytic activity across all concentrations, peaking at around 0.25 absorbance units at maximum concentrations(40 µg) (Fig. 9B). NNEV showed intermediate hemolytic activity, rising gradually with concentration and reaching ~ 0.3 absorbance units at 40 µg. NNSV concentrations between 5 and 10 µg (p-value 0.028) and NNEV concentrations between 10 and 20 µg (p-value 0.026) showed significance. Comparative activity between NNSV and NNEV across all concentrations among females showed no significant difference.

The hemolysis rate in males from Fig. 9A shows significantly reduced hemolytic activity compared to the female graph in Fig. 9B. The rate of hemolysis increased with the concentration. NNEV and NNSV induced minimal hemolysis in male erythrocytes, with tiny increments in absorbance across the concentration gradient (Fig. 9A). Among NNEVs, hemolytic activity between 10 and 20 µg showed a highly significant (p value 0.0001). Comparative activity between NNSV and NNEV across all concentrations among males showed no significant difference.

In females, DRSV exhibited the highest hemolytic activity at all concentrations tested (5–40 µg), with absorbance values reaching ~ 0.42 at 40 µg (Fig. 10B). DREV demonstrated almost equal hemolysis, with absorbance values closely trailing those of DRSV, indicating retention of hemolytic activity (Fig. 10B). Comparative activity between DRSV and DREV across all concentrations among females showed no significant difference.

In the case of males, DREV and DRSV again demonstrate the highest hemolytic activities among the four groups, though at far lower absolute absorbance values than in females (Fig. 10A). Comparative activity between DRSV and DREV across all concentrations among males showed no significant difference.

### ATPase and adpase assay

**Fig. 11:**
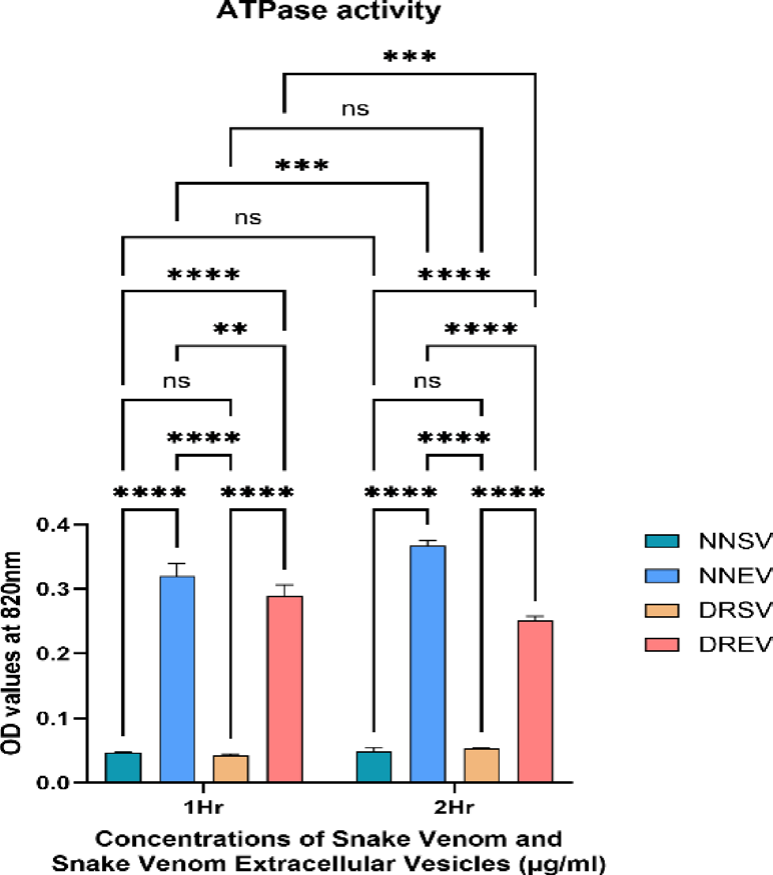
The graph illustrates the ATPase activity at varying concentrations of the four venom components: *Naja naja* venom (NNSV), *Naja naja* EVs (NNEV), *Daboia russelii* venom (DRSV), and *Daboia russelii* EVs (DREV). ATPase values were expressed in OD units. All assays were performed in triplicate, and the standard deviation is indicated by error bars. Statistical significance is indicated using the conventional asterisk system: *p < 0.05, **p < 0.001, ***p < 0.0001 and ****p < 0.0001.

**Fig. 12:**
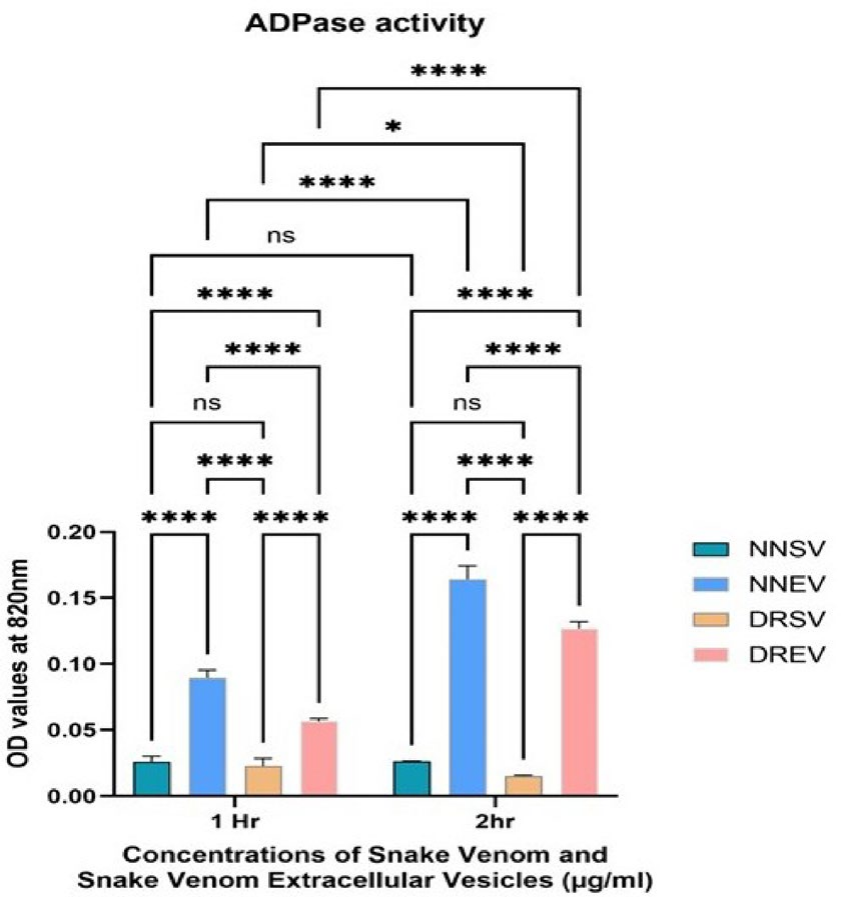
The graph illustrates the ADPase activity at varying concentrations of the four venom components: *Naja naja* venom (NNSV), *Naja naja* EVs (NNEV), *Daboia russelii* venom (DRSV), and *Daboia russelii* EVs (DREV). ADPase values were expressed in OD units. All assays were performed in triplicate, and the standard deviation is indicated by error bars. Statistical significance is indicated using the conventional asterisk system: *p < 0.05, **p < 0.001, ***p < 0.0001 and ****p < 0.0001.

NNSV, NNEV and DRSV showed an increase in ATPase activity from 1 h to 2 h, whereas DREV showed decreased activity compared to 1 h (Fig. 11).

#### ATPase activity at 1 h

NNEV exhibited the highest ATPase activity among all samples, with an absorbance close to 0.33. Observed highest activity between NNEV and DREV when compared to NNSV and DRSV, respectively (NNSV vs. NNEV p Value < 0.0001) (DRSV vs. DREV, p Value < 0.0001). DREV followed closely, showing a substantial ATPase activity (~ 0.29 absorbance units). NNSV and DRSV, the crude venoms, exhibited significantly lower ATPase activities than their derived EVs (Fig. 11).

#### ATPase activity at 2 h

ATPase activity in NNEV remained the highest and increased slightly to approximately 0.37 absorbance units, indicating sustained enzymatic function over time. DREV also showed a mild increase in activity (~ 0.31 absorbance), which showed statistical significance (NNSV vs. NNEV p Value < 0.0001) (DRSV vs. DREV, p Value < 0.0001). NNSV and DRSV saw modest gains in ATPase activity but remained very low compared to their EV counterparts (~ 0.06 and ~ 0.055, respectively).

Across all samples, ADPase activity increases from 1 to 2 h, suggesting hydrolytic activity progresses with time, as expected for enzymatic reactions. (Fig. 12).

#### ADPase activity at 1 h

NNEV shows the highest ADPase activity at 1 h, significantly greater than NNSV (p value < 0.0001). DREV also demonstrates appreciable activity compared to DRSV (p-value < 0.0001).

#### ADPase activity at 2 h

NNEV again has the highest activity, nearly double its 1-h level and a significant increase compared to NNSV (p value < 0.0001). DREV follows the same trend with an increase in activity (absorbance value ~ 0.12) when compared to DRSV (p-value < 0.0001). NNSV shows a moderate increase, while DRSV remains low.

Hence, both the venoms, NNEVs and DREVs showed considerably higher ATPase (NNSV vs. NNEV: p value < 0.001; DRSV vs. DREV: p value < 0.001) and ADPase activity (NNSV vs. NNEV: p value < 0.001; DRSV vs. DREV: p value < 0.001) at both time points when compared to NNSV and DRSV, respectively.

## Discussion

Snake venoms are intricate cocktails of bioactive molecules, comprising proteins, peptides, enzymes, and other small molecules, that exert diverse toxicological effects on prey or victims^17^. Studies have identified the presence of extracellular vesicles within various snake venoms^8^, suggesting that these vesicles may act as carriers for venom components, potentially modulating toxin biodistribution, stability, and activity^18^. The quality and characteristics of toxins encapsulated within EVs can differ from those of free (unencapsulated) toxins. Encapsulation within EVs can influence folding, stability, and even biological activity. Encapsulated proteins like the saporin toxin have demonstrated improved protection inside EVs and were efficiently delivered to target cells, retaining cytotoxic activity at doses where free toxin was ineffective. Using alkaline buffer loading methods, Zuppone et al. observed that the recombinant protein preserved both its structural integrity and functional activity upon encapsulation within EVs, supporting the notion that the vesicular microenvironment promotes proper protein folding and maintenance of bioactivity^19^.

As previously documented in prior investigations, we used venom concentrations in our tests^6^. These numbers are generally recognised as physiologically reasonable ranges for in vitro experiments, even if they might not match the amounts found after human envenomation. By selecting this range of concentrations, we created a controlled environment to directly compare the biological effects of extracellular vesicles and snake venom under similar circumstances.

PBS was used to isolate extracellular vesicles from snake venom. It is widely used due to its isotonic nature, but it may cause some damage, particularly during long-term storage or repeated freeze-thaw cycles. However, for short-term sample preparation, labelling, or molecular studies, PBS minimally impacts venom-derived EVs’ morphology and basic structure^20^.

For EVs produced from *Naja naja* and *Daboia russelii*, size discrepancies between NTA and TEM are predictable and explicable^21^. Particles look larger because of the hydration shell, surface proteins, adsorbed by the EVs, or protein corona^22^; Brownian-motion tracking also clusters transitory aggregation and protein complexes into apparent larger particles. NTA measures the hydrodynamic diameter in liquid. Staining can also conceal edges and lower the estimated diameter. TEM measures dehydrated, stained vesicles on a grid, frequently resulting in shrinkage and collapse. Proteases, lipoprotein-like complexes, and several low-molecular-weight toxins can co-isolate and artificially expand NTA distributions or form aggregates during isolation, which is one of the unique problems that snake venom introduces. Measured sizes are further shifted by instrument and analysis settings such as camera sensitivity, detection threshold, concentration for NTA; fixation, staining, drying in case of TEM^23,24^.

Western blotting showed the presence of CD63, TSG101 and HSP 90β in the isolated EVs of both *Naja naja* and *Daboia russelii* venom. TSG101 and CD63 are the canonical positive EV markers, per the MISEV guidelines^11^, whereas HSP90 is a negative EV marker, a well-documented general stress response chaperone^25,26^. No direct evidence exists that stress causes snakes to generate venom with higher amounts of HSP90. Nevertheless, mice in an experimental investigation were given a dose of *Daboia russelii* and *Naja kaouthia* venom, which displayed noticeably higher levels of inflammatory markers, such as HSP90^27^. HSP90 is a commonly reported co-isolated protein in extracellular vesicle preparations and has been classified as a “potential contaminant” in EV proteomics datasets^11^.

Among the enzymatic constituents of *Naja naja* venom, acetylcholinesterase (AChE) is particularly significant for its neurotoxicity. This enzyme catalyses the hydrolysis of acetylcholine (ACh) at synaptic junctions, interrupting neurotransmission and paralysis^28^. In contrast, viperid venoms, such as those from *Daboia russelii*, are primarily hemotoxic and exhibit comparatively low intrinsic AChE activity^29,30^. This difference reflects the broader functional divergence in venom strategies between elapids and viperids: neurotoxicity versus coagulopathy and haemorrhage in viperids. However, the present study indicates that EVs derived from *Daboia russelii* venom retain detectable AChE activity, albeit at lower levels than those from *Naja naja*. This finding implies that even in species where AChE is not a dominant toxin, EVs may serve to preserve and deliver enzymatic functions. The retention of enzymatic activity within venom-derived EVs may result from several factors.

Functionally, catalytically active AChE in venom-derived EVs carries significant implications for envenomation pathology. In *Naja naja*, EV-mediated transport of AChE may amplify neurotoxic effects by accelerating ACh hydrolysis at neuromuscular junctions. Conversely, in *Daboia russelii*, AChE within EVs, despite minimal activity in the whole venom, may play an ancillary or modulatory role, potentially influencing endothelial interactions, vascular tone, or platelet functions. Supporting this broader role of EVs, Gonçalves-Machado et al. demonstrated that Bothrops jararaca venom EVs are enriched in toxins such as metalloproteinases, PLA₂, and Serine proteases molecules, which, when they interact with Mammalian Cells, stimulate the local tissue environment and drive inflammatory responses. Such mechanisms may ultimately enhance the systemic impact of neurotoxins during envenomation^31^.

This study examined the dose-dependent effects of crude venom and venom-derived EVs from *Naja naja* and *Daboia russelii* on Prothrombin Time and Activated Partial Thromboplastin Time. Our findings elucidate the multifaceted role of soluble toxins and vesicle-packaged components in modulating coagulation pathways, with distinct activity patterns between species and fractions.

Extrinsic Pathway Disruption by Prothrombin Time; in DRSV & DREV, PT reflects the integrity of the extrinsic coagulation pathway (Factors VII, X, V, II). In our data, DRSV elicited a sharp increase in PT, reaching over 10 s at low concentrations (5 µg/mL), consistent with viperid venoms that carry both procoagulant and anticoagulant PLA_2_ and serine proteases. These toxins may activate Factor X or II in a consumptive manner, leading to venom-induced consumption coagulopathy (VICC)^32^.

DREV induced even more pronounced PT prolongation, especially at the highest concentration (40 µg/mL), suggesting that EVs encapsulate active components like anticoagulant PLA₂s and factor Xa inhibitors, and deliver them in a concentrated, stable form.

In case of NNSV and NNEV, PT elevation by NNSV was more gradual and dose-dependent, characteristic of elapid venoms with weaker hemotoxic effects and a propensity for neurotoxic activity. These venoms may contain PLA_2_s that inhibit thrombin or clotting factor Xa^33^. NNEV caused higher PT prolongation than NNSV, implying selective enrichment of these anticoagulant proteins within EV compartments.

Viperid venoms like DRSV contain both pro- and anticoagulant PLA_2_s and SVMPs, causing complex effects on PT. EVs may absorb or exclude procoagulant components, leading to differential net effects. Whereas Elapid venoms (NNSV) show milder extrinsic pathway interference, yet their EVs seem to concentrate factor Xa and thrombin inhibitors, thus amplifying activity.

Literature evidence strongly indicates that pelagic Daboia phospholipase A₂ (PLA₂) isoforms, with a focus on daboxin P, have high affinity for Factor X and its activated form (Xa), thus disorganising the prothrombinase complex and significantly prolonging coagulation at nanomolar concentrations^34^. Our results suggest EVs may serve as a delivery vehicle for such toxins, potentially enhancing pathogenicity during envenomation.

The capacity of EVs to extend PT longer than their whole venom indicates an advanced biological strategy by the snake to optimise systemic bleeding in predator or prey, supporting functional evolution of venom packaging into vesicular forms.

Intrinsic and Common Pathway Effects by Activated Partial Thromboplastin Time; in the case of DRSV and DREV, despite substantial PT prolongation, DRSV and DREV had diverging effects on aPTT. DRSV prolonged aPTT similarly to PT, indicating widespread consumption of clotting factors, including Factor IX and XI, which are typical of VICC. However, DREV displayed a dose-dependent reduction in aPTT from ~ 45 s at 5 µg/mL to ~ 25 s at 20 µg/mL, suggesting procoagulant activity when delivered via vesicles. This reorientation could be due to enrichment of Factor X activators or serine proteases in EVs.

NNSV and NNEV produced profound prolongation of aPTT (~ 85–90 s at ≥ 5 µg/mL), reflecting potent inhibition of the intrinsic coagulation pathway enzymes. This aligns with previous studies reporting elapid venom-derived PLA₂s and serine proteases that inhibit factors VIIIa, IXa, and/or prevent calcium–phospholipid complex formation^35^. The similarity between NNSV and NNEV aPTT profiles suggests that vesicle packaging preserves, rather than concentrates, anticoagulant effectors.

Understanding the hemolytic potential of snake venoms is essential for elucidating key components of envenomation pathology. Hemolysis can contribute to anaemia, renal injury, and systemic inflammatory complications in snakebite victims^36,37^.

In this study, we evaluated hemolytic rates induced by NNSV and DRSV alongside their venom-derived extracellular vesicles (NNEV and DREV), focusing on differences in activity, dose-dependence, gender, healthy individuals, and implications of EV-mediated enhancement or modulation of toxicity. Our results reveal an apparent dose-dependent increase across crude venom and EV samples. Female erythrocytes showed notably higher sensitivity, with absorbance values for DRSV and DREV at 40 µg. In contrast, male erythrocytes exhibited much lower hemolytic activity at 595 nm than females.

*Naja naja* and *Daboia russelii* display hemolytic properties; however, venom composition varies by species, explaining the differing magnitudes observed here. Elapid venoms such as *Naja naja* often contain direct cytotoxins and PLA₂s, causing immediate membrane disruption. In contrast, viperid venoms like *Daboia russelii* generally rely on indirect hemolysis, requiring cofactors such as phospholipids to exert their effect^37,38^. The higher hemolytic rates seen in DRSV for females thus likely result from a combination of potent PLA₂ isoforms and cofactors in the female erythrocyte membrane.

Critically, our data show that EV fractions preserved or amplified hemolytic activity, with DREV outperforming or nearly matching DRSV in female samples. Intriguingly, NNEV induced greater hemolysis than NNSV, especially in female donors, suggesting active concentration or stabilisation of hemolytic toxins within EVs. The data show significantly higher hemolysis in female versus male erythrocytes, regardless of venom source or fraction. While current literature on gender-specific hemolysis is limited, our results highlight the importance of considering gender as a critical biological variable in venom research.

Estrogen influences membrane fluidity and deformability by interacting with phospholipid bilayers and regulating the activity of membrane-bound enzymes. Experimental evidence demonstrates that estradiol can intercalate into lipid bilayers, altering packing density and decreasing membrane fragility^39,40^. Male RBCs, with relatively lower estrogen exposure, may exhibit higher susceptibility to venom-induced lysis due to less efficient protection against oxidative damage and altered lipid packing that facilitates toxin insertion^41^.

Adenosine triphosphatases (ATPases) catalyse the hydrolysis of ATP to ADP and inorganic phosphate, releasing energy vital for cellular processes such as ion transport, membrane potential maintenance, and intracellular signalling. In snake envenomation, ATPases, including Na^+^/K^+^-ATPases, are proposed to disrupt energy homeostasis, impair muscle function, and modulate ion gradients^42^.

However, the occurrence of ATPase activity within snake venoms and their EVs has not been thoroughly characterised. Our study tackles this gap by quantifying ATPase activity via absorbance (820 nm), comparing both crude venoms (NNSV, DRSV) and EV-associated fractions (NNEV, DREV) at 1 and 2 h.

A central finding is the marked enhancement of ATPase activity in venom-derived EVs, especially NNEV, which exhibited significantly higher activity than crude NNSV. Similarly, DREV displayed higher enzymatic activity than crude DRSV. This suggests that EVs are not passive byproducts but active nano-carriers of enzymes. EVs likely protect ATPases within their lipid bilayer, preventing degradation and concentrating enzymatic action. The observed kinetic increases (1 h to 2 h) imply that EV-based ATPases are enzymatically functional and active over sustained timeframes.

ATPases, particularly the Na^+^/K^+^ pumps, are essential for cellular homeostasis, maintaining electrochemical gradients. Proteins in *Naja* venom can inhibit these ATPases; studies have shown *Naja mossambica* venom contains components that disrupt Na^+^/K^+^-ATPase activity in rat brain membranes^43^.

Elevated EV-carried ATPase activity may disrupt host cell ATP balance, impair muscle function or nerve conduction due to altered ion gradients, and enhance venom potency by leveraging endogenous ATP stores and membrane disruption.

The marked ATPase activity in NNEV surpasses that in DREV, reflecting differences in venom composition and EV biochemistry. Elapid venoms traditionally contain neurotoxins and PLA₂s, with some exhibiting ATPase inhibitory effects, whereas viperid venoms focus on hemotoxins and coagulopathic toxins; their EVs appear to carry moderate ATPase activity enough to mediate cellular uptake. Hence, Cobra EVs may be fine-tuned to disrupt neuronal or muscular cells via ATP depletion. Viper EVs might facilitate haematological symptoms through milder ATP disturbance.

NNEV and DREV showed sustained and slightly enhanced activity from 1 to 2 h, while crude venoms remained low. This underscores that EV-stabilised ATPases are kinetically robust, resisting degradation over time.

Adenosine diphosphatase (ADPase), called apyrase, plays a critical role in hemostasis by hydrolysing extracellular ADP to AMP and inorganic phosphate (Pi), effectively inhibiting ADP-mediated platelet aggregation. Snake venoms have been recognised sources of such ecto-nucleotidases, which include ADPase and related enzymes like 5′-nucleotidase (CD73-like) and nucleoside phosphodiesterases^15,43^. ADPase activity contributes to venom-induced coagulopathy and immune modulation^44^.

NNEV exhibited the highest ADPase activity, far exceeding that of crude NNSV. DREV showed moderate but significant ADPase activity, surpassing DRSV. These results underscore EVs as active toxin carriers, extending venom functionality beyond soluble toxins^31,45^.

The potent ADPase activity of NNEV highlights the venom’s neurotoxic profile, complemented by antiplatelet activity, possibly preventing thrombosis at bite sites and aiding venom diffusion. Several cobra species express apyrases or CD39-like nucleotide hydrolases^46^, and purified cobra ADPase enzymes inhibit ADP-induced aggregation at low concentrations^47^. The EV-based amplification of this effect aligns with prey immobilisation strategies involving vascular and hemostatic disruption.

Viperid venoms traditionally induce coagulopathy through metalloproteinases and serine proteases^48^. The presence of ADPase in DREV suggests an integrated hemostatic disruption strategy, facilitating platelet disaggregation and enhancing hemorrhagic symptoms.

By eliminating ADP, a primary platelet activator, snake venom ADPase prevents aggregation and thrombosis. Our robust EV activity data imply that inhibition was intensified when enzymes were delivered via vesicles, potentially leading to prolonged bleeding and delayed hemostasis.

Hydrolysis of ADP to AMP by ADPase and further to adenosine may have anti-inflammatory and immunomodulatory roles^49^. Adenosine promotes vasodilation and immunosuppression, potentially enhancing venom spread and suppressing local immunity^50^. EVs may thus serve as biochemical modulators, not just mechanical disruptors. These enzymes may also persist post-antivenom due to EV protection, contributing to chronic or delayed bleeding complications.

Thus, the lipid bilayer of EVs could protect encapsulated enzymes from proteolytic degradation, maintaining their structural and functional integrity^51^. Additionally, EVs might enhance the local concentration of enzymes at target sites and facilitate cell-specific delivery, thereby increasing the biological efficacy of venom component^18^. Future research should focus on systemic distribution, organ-specific targeting, and pathophysiological significance of venom EVs during envenoming and extrapolate these findings to in vivo models. In addition to evaluating the potential of EV-targeted therapies in enhancing antivenom efficacy and therapeutic outcomes, animal studies might look more closely at their role in neurotoxicity, coagulopathy, and hemolysis.

## Conclusion

Our research demonstrates that extracellular vesicles isolated from the venoms of *Naja naja* and *Daboia russelii* are not mere passive transporters but active facilitators of envenomation. These EVs possess high enzymatic activities of AChE, ATPase, and ADPase and regulate major physiological processes like coagulation, hemolysis, and metabolic breakdown. Specifically, EVs from *Naja naja* display acetylcholinesterase activity comparable to that of the whole venom, but exhibit markedly higher ATPase activity, aligning with their neurotoxic activity profile. *Daboia russelii* EVs augment or reverse hemostatic activity, albeit with lesser enzymic concentrations in crude venom. Both EVs are dose-dependent, hemolytically active with gender-differentiated susceptibility, a feature evincing the necessity for a sexual therapeutic strategy. EVs maintain enzymatic integrity, stabilise toxins, possibly by membrane protection, and focus their actions on cell surfaces through membrane fusion. This multi-functionality highlights their function in venom potency and complexity augmentation. Traditional antivenoms, which primarily target soluble toxins, may be insufficient; innovative therapeutic approaches should consider EV-disrupting agents or vesicle-specific antibodies. Our findings highlight the need for intense venom EVs research, especially considering EVs in antivenom development and envenoming models. Recognising EVs as dynamic, modular toxin platforms offers new insights into venom evolution, disease mechanisms, and potential therapeutic strategies, thereby broadening current perspectives in venom biology and snakebite treatment.

## Supplementary Information

Below is the link to the electronic supplementary material.

Supplementary Material 1

Supplementary Material 2

Supplementary Material 3

## Data Availability

The datasets generated and/or analysed during the current study are available from the corresponding authors on reasonable request.
