# Supplementary figures and images for: Unveiling the nanotoxicology of snake venoms through functional and biochemical characterization of extracellular vesicles from Naja naja and Daboia russelii
Source: Sci Rep. 2025 Nov 29;15:42860. doi: 10.1038/s41598-025-27041-6 (PMC12669237; doi:10.1038/s41598-025-27041-6)

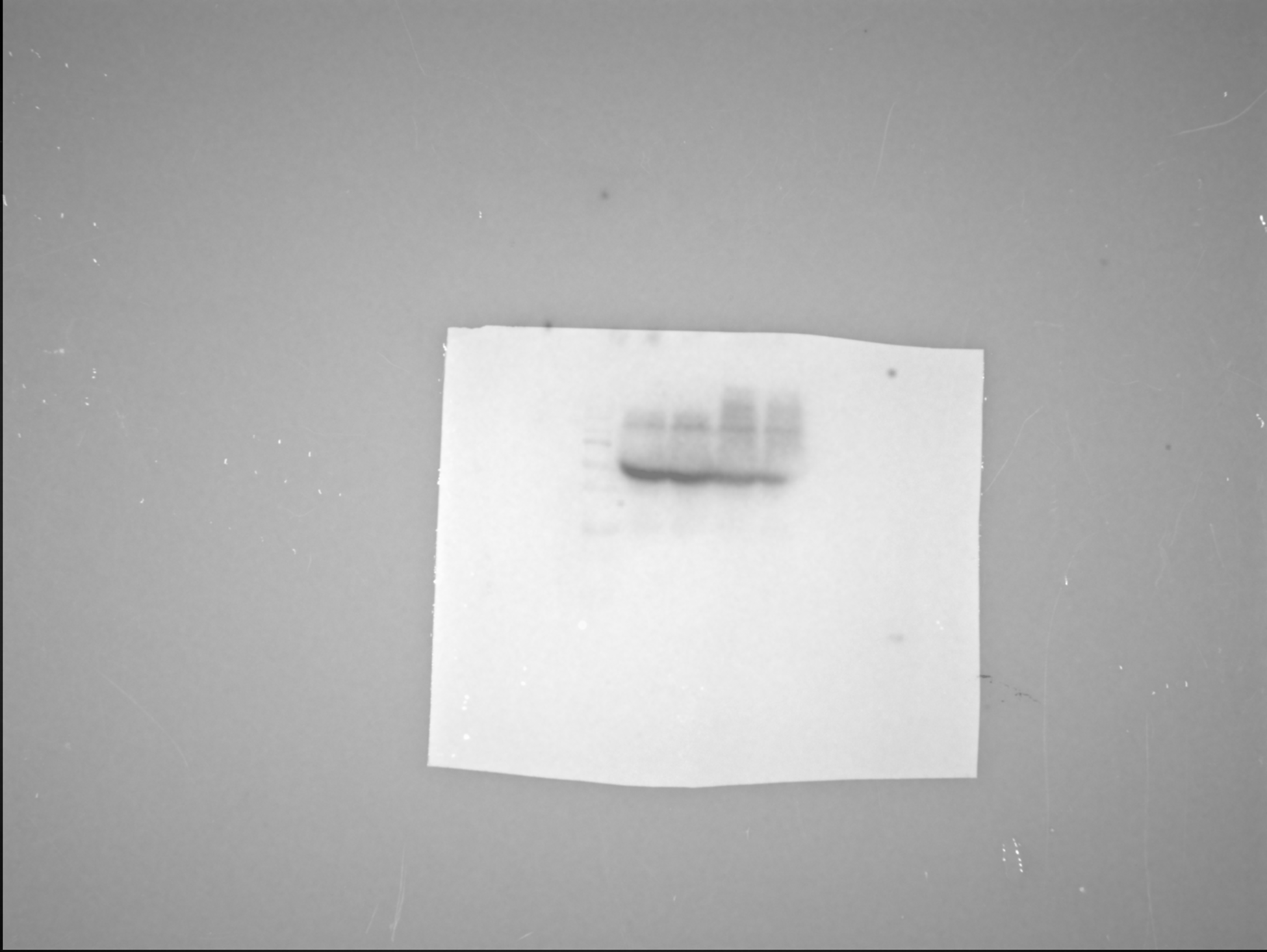

Supplement: Supplementary file 1 — Supplementary Material 1 [file 41598_2025_27041_MOESM1_ESM.tif]

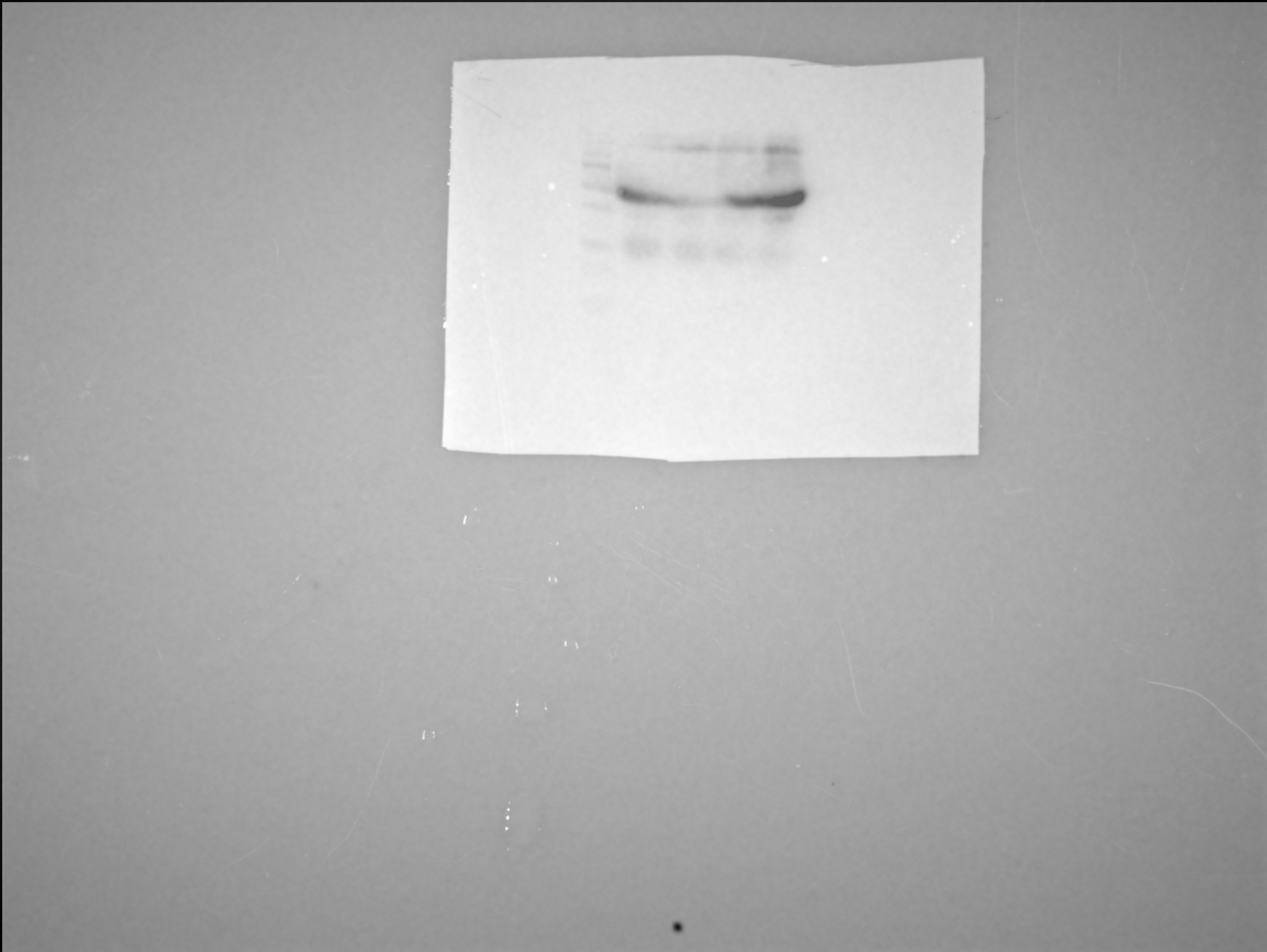

Supplement: Supplementary file 2 — Supplementary Material 2 [file 41598_2025_27041_MOESM2_ESM.tif]

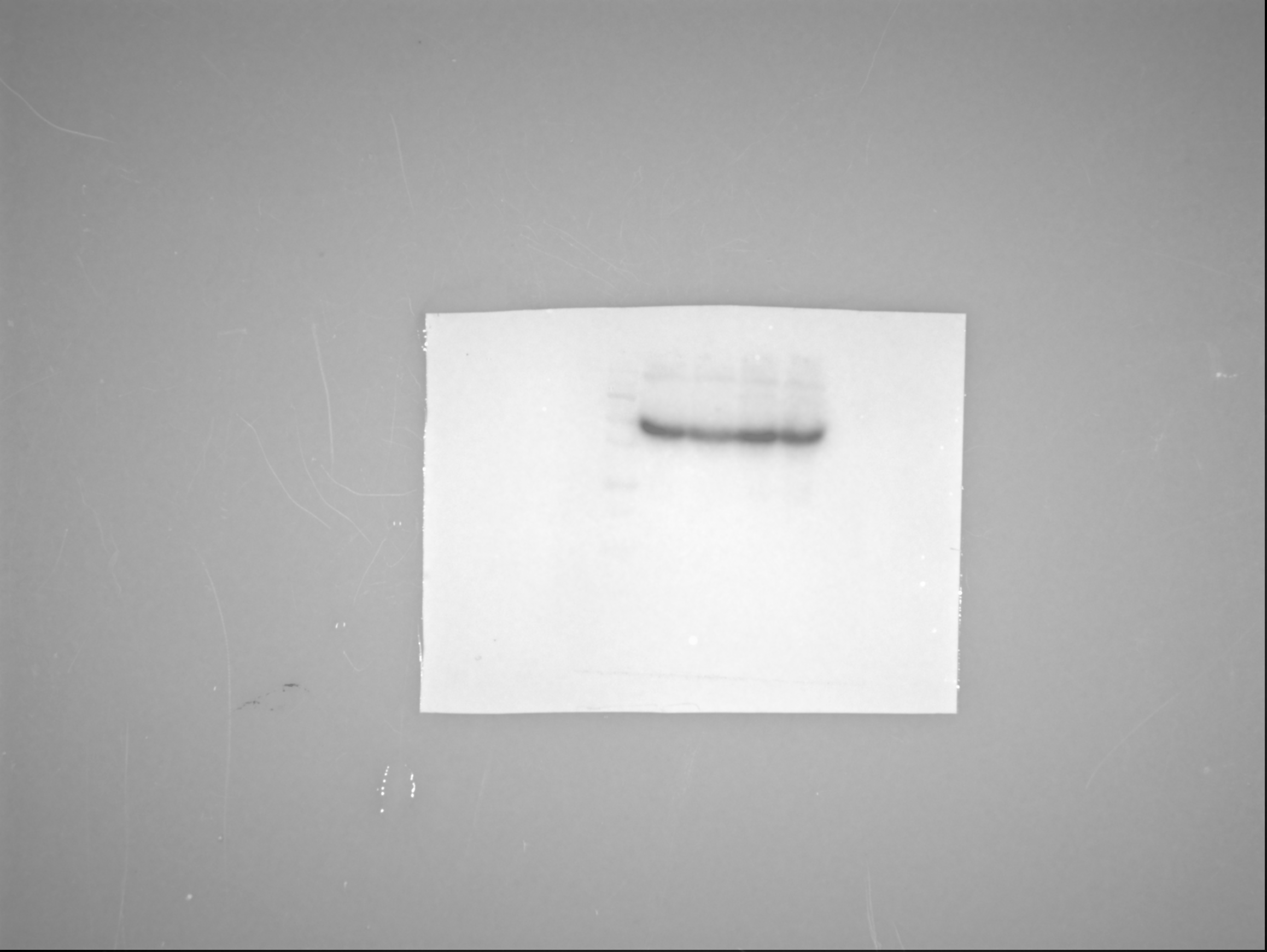

Supplement: Supplementary file 3 — Supplementary Material 3 [file 41598_2025_27041_MOESM3_ESM.tif]
